# Changes in DNA Methylation of *Clock* Genes in Obese Adolescents after a Short-Term Body Weight Reduction Program: A Possible Metabolic and Endocrine Chrono-Resynchronization

**DOI:** 10.3390/ijerph192315492

**Published:** 2022-11-22

**Authors:** Antonello E. Rigamonti, Valentina Bollati, Chiara Favero, Benedetta Albetti, Diana Caroli, Alessandra De Col, Silvano G. Cella, Alessandro Sartorio

**Affiliations:** 1 Department of Clinical Sciences and Community Health, University of Milan, 20129 Milan, Italy; 2 EPIGET Lab, Department of Clinical Sciences and Community Health, University of Milan, 20122 Milan, Italy; 3 Occupational Health Unit, Fondazione IRCCS Ca’ Granda Ospedale Maggiore Policlinico, 20122 Milan, Italy; 4 Istituto Auxologico Italiano, Istituto di Ricovero e Cura a Carattere Scientifico (IRCCS), Experimental Laboratory for Auxo-Endocrinological Research, 28824 Verbania, Italy; 5 Istituto Auxologico Italiano, Istituto di Ricovero e Cura a Carattere Scientifico (IRCCS), Experimental Laboratory for Auxo-Endocrinological Research, 20145 Milan, Italy

**Keywords:** obesity, *clock* genes, DNA methylation, chronobiology, body weight reduction program, childhood, cardiometabolic outcomes, HPA function

## Abstract

Circadian rhythms are generated by a series of genes, collectively named *clock* genes, which act as a self-sustained internal 24 h timing system in the body. Many physiological processes, including metabolism and the endocrine system, are regulated by *clock* genes in coordination with environmental cues. Loss of the circadian rhythms has been reported to contribute to widespread obesity, particularly in the pediatric population, which is increasingly exposed to chronodisruptors in industrialized society. The aim of the present study was to evaluate the DNA methylation status of seven *clock* genes, namely *clock*, *arntl*, *per1-3* and *cry1-2*, in a cohort of chronobiologically characterized obese adolescents (n: 45: F/M: 28/17; age ± SD: 15.8 ± 1.4 yrs; BMI SDS: 2.94 [2.76; 3.12]) hospitalized for a 3-week multidisciplinary body weight reduction program (BWRP), as well as a series of cardiometabolic outcomes and markers of hypothalamo–pituitary–adrenal (HPA) function. At the end of the intervention, an improvement in body composition was observed (decreases in BMI SDS and fat mass), as well as glucometabolic homeostasis (decreases in glucose, insulin, HOMA-IR and Hb1Ac), lipid profiling (decreases in total cholesterol, LDL-C, triglycerides and NEFA) and cardiovascular function (decreases in systolic and diastolic blood pressures and heart rate). Moreover, the BWRP reduced systemic inflammatory status (i.e., decrease in C-reactive protein) and HPA activity (i.e., decreases in plasma ACTH/cortisol and 24 h urinary-free cortisol excretion). Post-BWRP changes in the methylation levels of *clock*, *cry2* and *per2* genes occurred in the entire population, together with hypermethylation of *clock* and *per3* genes in males and in subjects with metabolic syndrome. In contrast to the pre-BWRP data, at the end of the intervention, cardiometabolic parameters, such as fat mass, systolic and diastolic blood pressures, triglycerides and HDL-C, were associated with the methylation status of some *clock* genes. Finally, BWRP induced changes in *clock* genes that were associated with markers of HPA function. In conclusion, when administered to a chronodisrupted pediatric obese population, a short-term BWRP is capable of producing beneficial cardiometabolic effects, as well as an epigenetic remodeling of specific *clock* genes, suggesting the occurrence of a post-BWRP metabolic and endocrine chronoresynchronization, which might represent a “biomolecular” predictor of successful antiobesity intervention.

## 1. Introduction

Owing to the changeable nature of environmental conditions in living beings, including the daily or seasonal availability of food, a complex system of chronobiological regulation has been evolved. This system imparts circadian rhythmicity to a variety of biological phenomena, including hormonal secretion, consummatory behaviors and metabolism [1].

The suprachiasmatic nucleus (SCN), located at the hypothalamic level, is monosinaptically connected to photosensitive ganglion neurons that, within the retina, detect the light/dark cycle from the external environment, with ensuing internal chronosynchronization [2]. Therefore, SCN has been acknowledged to act as master pacemaker, upon which the circadian rhythmicity of many peripheral organs depends, including pituitary and pineal gland, liver and adipose tissue, which, when disconnected from hierarchical SCN control, maintain a proper chronobiological autonomy [3].

Specific genes collectively known as *clock* genes have been identified for a long time. In particular, they encode nuclear transcriptional factors or coactivator/repressors that modulate the expression of a multitude of genes (about 20% of all genes present in the nucleus of a eukaryotic cell) [1].

In particular, CLOCK (circadian locomotor output cycles kaput) and ARNTL (aryl hydrocarbon receptor nuclear translocator-like, which is alternatively named BMAL1 (brain- and muscle-ANRT-like protein) are (transcriptionally) positive nuclear factors taking part in a chronobiological feedback loop in which NPAS2 (name derived from PERARNT-SIM protein-2) is also engaged. The CLOCK-ARNTL heterodimer binds to a specific DNA sequence named E-box, which is present within the promoter region of some target genes, which, in turn, regulate the transcription of other *clock* genes, such as the PER (period genes) family and the CRY (cryptochrome genes) family. The protein products of *per* and *cry* genes dimerize and are translocated into the nucleus. The transcription of genes encoding the repressors is blocked when the levels of PER-CRY are sufficient to antagonize the positive effect of CLOCK-ARNTL, with subsequent inhibition of the CLOCK-ARNTL-dependent transcription.

Other nuclear receptors and coactivators/repressors, such as peroxisome proliferator-activated receptors (PPARs) and PPAR-γ coactivator 1α (PGC 1α), have been recognized as modulators of *arntl*/ARNTL and *clock*/CLOCK [4]. Taking into account the pre-eminent role exerted by PPARs in the regulation of glucose and lipid metabolism in the liver, muscles and adipose tissue, as well as in the proliferation/differentiation of adipocyte progenitors [5,6], the strong interrelationships among chronobiology, obesity and metabolic syndrome are not surprising [7].

In this context, *clock* mutant mice, as other genetically modified (knockout and transgene) animal models in *clock* genes, are obese due to hypertrophied visceral adipose tissue, together with liver steatosis, hyperglycemia, hyperinsulinemia, hypertriglyceridemia and hyperleptinemia, which, overall, resemble (human) metabolic syndrome [8,9].

In humans, a considerable number of epidemiological studies have demonstrated the existence of a strong association of obesity with known chronodisruptors, including working night shifts, long-lasting exposure to artificial light, sleep deprivation, nocturnal snacking, irregular daily eating times, etc. [10]. Most chronodisruptors are typically present in the so-called nocturnal chronotype, which is becoming increasingly prevalent in the modern civilized population living in the urban centers [11].

Many endocrine axes, such as the hypothalamo–pituitary–adrenal (HPA) axis, show a typical circadian secretory pattern [12]. In particular, secretion of corticotropin (ACTH) and cortisol is generally higher in the morning and falls throughout the day [13]. There is strong evidence that in obese subjects, accumulation of visceral adipose tissue and dysmetabolism are associated with a chronodisruption of HPA function [14,15]. Furthermore, even in normal-weight subjects, many chronodisruptors negatively affect HPA function (e.g., psychological stress, jet lag or working nocturnal shifts) [16]. As *clock* gene expression and diet rhythmicity have been demonstrated to be regulated by glucocorticoid receptors [17,18], the existence of a link among *clock* genes, the HPA axis and obesity cannot be ruled out [19].

Obesity is a multifactorial disease derived from a combination of environmental factors (e.g., hypercaloric diet and sedentary lifestyle) with a polygenetic predisposition [20]. In recent years, an epigenetic dysregulation has been documented in obesity and its cardiometabolic comorbidities, particularly alterations in DNA methylation status, such as hypo/hypermethylation of *clock* genes [21,22]. DNA methylation is an epigenetic mechanism involving the transfer of a methyl group to the C5 position of the cytosine to form 5-methylcytosine [23]. DNA methylation occurring in gene promoters is mostly associated with the silencing of gene expression through the recruitment of proteins involved in gene repression or the inhibition of transcription factor binding to DNA [24]. The pattern of DNA methylation is continuously modified by external stimuli, in a dynamic process involving both de novo DNA methylation and demethylation [25]. This plasticity also involves *clock* genes [26].

In this regard, diet patterns have been reported to change the methylation status of CpG sequences within *clock* genes [27,28]. Moreover, some protein products of *clock* genes have been reported to be directly involved in the biochemical mechanisms underlying epigenetics (e.g., CLOCK as histone-acetyltransferase) [29]. Accordingly, in a study carried out in obese adults, DNA methylation of *clock*, *bmal*1 (or *arntl*) and *per2* genes was shown to be associated with anthropometric and biochemical parameters that are related to obesity, metabolic syndrome and weight loss after a program of metabolic rehabilitation [30].

Based on previous considerations, with the spread of obesity and the nocturnal chronotype in the pediatric population [31], the aim of the present clinical study was to evaluate, in a context of chronobiological parameters, a cohort of obese adolescents hospitalized for a short-term (3-week) body weight reduction program (BWRP). In particular, we determined the DNA methylation of a series of *clock* genes (i.e., *clock*, *arntl*, *cry*1-2 and *per1*-3), which were associated with clinical, anthropometric and biochemical parameters in peripheral leukocytes collected before and after the 3-week BWRP, stratifying the study population by sex and metabolic syndrome. Furthermore, chronotype and HPA function were evaluated.

Whereas BWRP-induced cardiometabolic benefits are well-documented in the medical literature [32,33,34], our hypothesis was that a short-term BWRP is capable of modifying the DNA methylation pattern of *clock* genes, providing novel insight into the relationships between epigenetics and chrononutrition, with the possibility of therapeutically resynchronizing chronodisrupted obese subjects through easy lifestyle modifications, including diet and exercise [27].

## 2. Materials and Methods

### 2.1. Subjects

A set of adolescents was selected from a patient population admitted to the Division of Auxology of the Istituto Auxologico Italiano, Piancavallo (VB), Italy, for a 3-week in-hospital multidisciplinary BWRP.

The inclusion criteria were individuals of both sexes aged ≤ 18 yrs with a body mass index (BMI) (or BMI deviation standard score, BMI-SDS) > 97th percentile according with age- and sex-specific Italian growth charts [35], with or without metabolic syndrome (see below for its definition). The exclusion criteria were: (1) secondary causes of obesity (e.g., Prader–Willi syndrome, steroid–induced or medication-induced obesity); (2) individuals with systolic blood pressure (SBP) ≥ 180 mmHg and diastolic blood pressure (DBP) ≥ 110 mmHg; (3) cardiovascular disease clinically evident in the previous 6 months; (4) psychiatric, neurological, osteomuscular or rheumatologic diseases that limit the ability to undertake a (standard) 3-week in-hospital period of metabolic rehabilitation, including physical activity (see below for details); and (5) individuals (and/or their parents) who refused to sign the consent form.

The study protocol was approved by the Ethical Committee of the Istituto Auxologico Italiano, IRCCS, Milan, Italy (research project code: 01C922; acronym: GENICLOCK); the protocol was explained to the patients and/or their parents, who gave their written informed consent.

### 2.2. Body Weight Reduction Program (BWRP)

The BWRP consisted of a 3-week multidisciplinary in-hospital metabolic rehabilitation, entailing an energy-restricted diet, physical rehabilitation (moderate aerobic activity), psychological counseling and nutritional education. The amount of energy to be provided by diet was calculated by subtracting approximately 500 kcal from the measurement of resting energy expenditure (REE), which was determined after an overnight fast by means of open-circuit, indirect computerized calorimetry (Vmax 29, Sensor Medics, Yorba Linda, CA, USA) with a rigid, transparent, ventilated canopy. In terms of macronutrients, the diet contained approximately 21% proteins, 53% carbohydrates and 26% lipids; the daily estimated water content was 1000 mL, whereas the estimated salt content was 1560 mg Na^+^, 3600 mg K^+^ and 900 mg Ca^2+^. Extra water intake of at least 2000 mL/day was encouraged.

The physical activity program consisted of 5 days per week training, including (i) 1 h dynamic aerobic standing and floor exercise with arms and legs at moderate intensity and under the guidance of a therapist and (ii) either 20–30 min cycloergometer exercise at 60 W or 3–4 km outdoor walking on flat terrain according to individual capabilities and clinical status.

The subjects also underwent a psychological counseling program consisting of two or three sessions per week of individual and/or group psychotherapy performed by clinical psychologists. Furthermore, lectures, demonstrations and group discussions with or without a supervisor took place daily.

### 2.3. Anthropometric Measurements

A scale with a stadiometer was used to determine height and weight (Wunder Sa.Bi., WU150, Trezzo sull’Adda, Italy). Waist circumference (WC) was measured with a flexible tape in standing position, halfway between the inferior margin of the ribs and the superior border of the crista, whereas hip circumference (HC) was measured at the largest parts around the buttocks. Body composition was measured by bioimpedance analysis (Human-IM Scan, DS-Medigroup, Milan, Italy) after 20 min of supine resting and in accordance with the international guidelines [36]. BMI, fat mass (FM) and fat-free mass (FFM) were determined in all subjects.

### 2.4. Metabolic, Biochemical and Hormonal Evaluation

Blood samples (about 10 mL) were collected at around 8:00 AM after an overnight fast at the beginning of the BWRP at T0 and at the end (i.e., 21st day, T1). A further blood sample was drawn at 15:00 (only for evaluation of HPA axis). Total cholesterol (T-C), high-density lipoprotein cholesterol (HDL-C), low-density lipoprotein cholesterol (LDL-C), triglycerides (TGs), glucose, insulin, C-reactive protein (CRP), cortisol (at 08:00 AM and 03:00 PM, i.e., cortisol-8AM and cortisol-3PM, respectively) and ACTH (at 08:00 AM and 03:00 PM, i.e., ACTH-8AM and ACTH-3PM, respectively) were measured. The 24 h urine was collected (at T0 and T1) for the determination of free cortisol excretion.

Colorimetric enzymatic assays (Roche Diagnostics, Monza, Italy) were used to determine serum T-C, LDL-C, HDL-C and TG levels. The sensitivities of the assays were 3.86 mg/dL [1 mg/dL = 0.03 mmol/L], 3.87 mg/dL [1 mg/dL = 0.03 mmol/L], 3.09 mg/dL [1 mg/dL = 0.03 mmol/L] and 8.85 mg/dL [1 mg/dL = 0.01 mmol/L], respectively.

The serum glucose level was measured by the glucose oxidase enzymatic method (Roche Diagnostics, Monza, Italy). The sensitivity of the method was 2 mg/dL [1 mg/dL = 0.06 mmol/L].

The serum insulin concentration was determined by a chemiluminescent immuno-metric assay using a commercial kit (Elecsys Insulin, Roche Diagnostics, Monza, Italy). The sensitivity of the method was 0.2 µIU/mL [1 µU/mL = 7.18 pmol/L].

The intra- and interassay coefficients of variation (CVs) were 1.1% and 1.6% for T-C, 1.2% and 2.5% for LDL-C, 1.8% and 2.2% for HDL-C, 1.1% and 2.0% for TG, 1.0% and 1.3% for glucose, and 1.5% and 4.9% for insulin.

CRP was measured using an immunoturbidimetric assay (CRP RX, Roche Diagnostics GmbH, Mannheim, Germany). The sensitivity of the method was 0.03 mg/dL.

Cortisol (in plasma and urine) was detected using a commercial ELISA kit (IBL-Hamburg Gmbh, Hamburg, Germany). Intra- and interassay CVs for this assay were <8.0% and <15%, respectively. The sensitivity of the method was 1.5 μg/L.

ACTH was determined by chemiluminescent enzyme immunoassay “ECLIA” Elecsys ACTH (Cobas, Roche diagnostics Gmbh, Mannheim, Germany). Intra- and interassay CVs for this analytical method were <3.2% and <5.4%, respectively. The sensitivity of the method was 1.00 ng/L.

For each patient, we also calculated the homeostatic model assessment of insulin resistance (HOMA-IR) according to the following formula: (insulin [μIU/mL] × glucose [mmol/L])/22.5.

### 2.5. Evaluation of Blood Pressure

Blood pressure was measured on the right arm using a sphygmomanometer with an appropriate pediatric cuff size, with the subject in a seated position and relaxed condition. The procedure was repeated three times at 10 min intervals; the means of the three values for SBP and DBP were recorded.

### 2.6. Definition of Metabolic Syndrome

According to the IDF (International Diabetes Federation) criteria for diagnosis of metabolic syndrome in children and adolescents [37], our patients were considered positive for the presence of metabolic syndrome if they had abdominal obesity (WC ≥ 90th percentile [38] for ages <16 years and ≥94 cm for males and ≥80 cm for female for ages >16 years) plus two or more of the following factors: (i) increased TG level ≥ 150 mg/dL (1.7 mmol/L) for ages < 16 years and the same cutoff or specific treatment for this lipid abnormality for ages > 16 years, (ii) reduced HDL-C < 40 mg/dL (1.03 mmol/L) for males and females for ages < 16 years and <40 mg/dL for males and <50 mg/dL (1.29 mmol/L) for females or specific treatment for this lipid abnormality for ages > 16 years, (iii) increased BP: SBP ≥ 130 mmHg or DBP ≥ 85 mmHg for ages < 16 years and the same cutoff or treatment of previously diagnosed hypertension for ages > 16 years and (iv) increased fasting glucose concentration ≥ 100 mg/dL (5.6 mmol/L) or previously diagnosed type 2 diabetes mellitus for all ages.

### 2.7. Evaluation of Chronodisruptors

Before BWRP (basal condition, T0), each subject was chronobiologically characterized by the following tests.

#### 2.7.1. Sleep Duration

Habitual sleep time was estimated by a questionnaire containing the following questions:

(1). During week days: how many hours (and minutes) do you usually sleep?
(2). During weekend days: how many hours (and minutes) do you usually sleep?

A total weekly sleep score was calculated as [(min weekdays × 5) + (min weekend days × 2)]/7 [39].

#### 2.7.2. Eveningness

Eveningness was assessed by the Horne and Ostberg [40] questionnaire to assess morningness–eveningness (MEQSA, morningness–eveningness questionnaire self-assessment).

This questionnaire establishes not only a (quantitative) total score, but also five behavioral categories, which, in the present study, were reduced to three ones: definitively morning types (score = 70–86), intermedial types (score = 31–69), and definitively evening types (score = 16–30).

#### 2.7.3. Sleepiness

The Cleveland Adolescent Sleepiness Questionnaire (CASQ), a brief, self-completed instrument to measure excessive daytime sleepiness specifically developed for adolescents was administered [41].

The total CASQ score, as well as sleepiness and alertness scores, was used for statistical analysis.

### 2.8. DNA Extraction and Bisulfite Treatment

Another 7 mL of whole blood was collected into EDTA tubes from each participant (at T0 and T1). After centrifuging the blood tubes at 1200× *g* for 15 min to separate plasma, buffy coat and erythrocytes, genomic DNA was extracted from the buffy-coat fraction using a Wizard Genomic DNA Purification Kit (Promega; Madison, WI, USA) according to the manufacturer’s instructions. The concentration of the purified DNA was measured using a NanoDrop-1000 spectrophotometer (Thermo Fisher Scientific; Waltham, MA, USA). The DNA samples were plated at a concentration of 25 ng/µL in 96-well plates and treated with sodium bisulfite using an EZ-96 DNA Methylation-Gold™ kit (Zymo Research; Irvine, CA, USA) following the manufacturer’s instructions. After elution, each DNA sample was divided into 10 µL aliquots using a Microlab STAR Automated Liquid Handling Workstation (Hamilton Company; Reno, NV, USA), and the plates were stored at −80 °C until use.

### 2.9. DNA Amplification and Pyrosequencing

DNA methylation was analyzed via previously published methods with minor changes [42,43]. Briefly, 10 µL of bisulfite-treated template DNA was added to 25 µL of GoTaq Hot Start Green Master Mix (Promega), 1 µL of forward primer (10 µM) and 1 µL of 5-t-end-biotinylated reverse primer (10 µM) to set up a 50 µL PCR reaction [44]. PCR cycling conditions and primer sequences are reported in Supplementary Table S1.

The biotin molecule at the 5 t extremity of reverse primers was exploited to isolate a single DNA filament, which was subsequently used as a template for pyrosequencing. The whole procedure was performed using a Pyromark^®^ Gold Q96 kit (QIAGEN GmbH, Hilden, Germany). Briefly, after incubating 15 µL of PCR product with streptavidin–Sepharose HP beads (Amersham BioSciences Ltd., Little Chalfont, UK), the biotin-labeled single-stranded DNA was purified, washed, denatured with 0.2 M NaOH and washed again using a Pyrosequencing Vacuum Prep Tool (QIAGEN). After elution, the purified DNA filament was briefly incubated in an annealing mix containing the sequencing primer (0.3 µM), and the plates were then heated up to 85 °C. Pyrosequencing was performed with a PyroMark MD system (QIAGEN). CpG sites were queried within the promoter regions of the following genes: *arntl*, *clock*, *cry*1, *cry*2, *per1*, *per2* and *per3*.

Quantitative analysis of the methylation level at individual CpG positions within each gene’s promoter region was carried out using Pyro Q-CpG software (Biotage, Uppsala, Sweden), which indicates the percentage of methylated cytosines among the total number of cytosines (5-methyl-cytosine + unmethylated cytosines) at each CpG site of interest. Measures of individual CpGs were averaged and used in the statistical analysis. Every sample was tested twice for each gene to guarantee the reproducibility of the experimental setting.

### 2.10. Statistical Analysis

Pre- and post-BWRP demographic, lifestyle, biochemical and clinical characteristics were compared as continuous variables using linear regression models for paired data. Categorical data were compared with a McNemar test for paired data.

Linear mixed regression models for paired data were used to evaluate associations between time (pre/post BWRP) and *clock* gene methylation.

We applied linear mixed regression models for paired data to evaluate the modifier effect of gender and metabolic syndrome (yes/no) on associations between time (pre/post BWRP) and biochemical/clinical characteristics or methylation of *clock* genes, classified as continuous variables; for categorical variables, we applied the McNemar test for paired data.

Linear regression models were applied to evaluate the associations between methylation of *clock* genes and the lifestyle, biochemical and clinical characteristics measured pre or post BWRP. Models were adjusted for sex, smoking habits and BMI SDS.

Owing to the large number of comparisons, we used a multiple comparison method based on Benjamini–Hochberg false-discovery rate (FDR) to calculate the FDR *p*-value.

The statistical analyses were performed using SAS software (version 9.4, SAS Institute, Milan, Italy). *p*-values below 0.05 were considered statistically significant.

## 3. Results

### 3.1. Subject Characteristics

Forty-five obese adolescents (F/M: 28/17; mean age ± SD: 15.8 ± 1.4 yrs; BMI SDS: 2.94 [2.76; 3.12]) were recruited and, having completed the 3 weeks of BWRP, included in the study. Table 1 summarizes the demographic, lifestyle, biochemical and clinical characteristics of the entire population in basal condition (at T0, i.e., before the BWRP) and at the end of the intervention (at T1, i.e., after the 3-week BWRP).

### 3.2. Effects of the BWRP in the Entire Population

At the end of the BWRP, among the entire population, BMI or BMI SDS significantly decreased (vs. BMI or BMI SDS at T0, *p* < 0.0001). Changes in body composition were produced by the BWRP (pre vs. post BWRP); in particular, the BWRP significantly reduced both FM % and FFM kg (*p* < 0.0001), with an unchanged post-BWRP value of REE. The BWRP also produced beneficial metabolic effects (pre-vs. post BWRP); in particular, there were significant decreases in glucose (*p* < 0.0001), insulin (*p* < 0.0001), HOMA-IR (*p* < 0.0001), HbA1c (*p* < 0.0001), T-C (*p* < 0.0001), LDL-C (*p* < 0.0001), HDL-C (*p* < 0.0001), NEFA (*p* < 0.0001) and TG (*p* < 0.0001). An improvement in cardiovascular function was also evident at the end of the BWRP (pre vs. post BWRP); significant reductions in SBP (*p* < 0.0001), DBP (*p* < 0.0001) and HR (*p* 0.0171) were observed. The BWRP significantly reduced some markers of systemic inflammation (pre vs. post BWRP), such as CRP (*p* < 0.0001). Finally, despite the post-BWRP cardiometabolic improvements, the prevalence of metabolic syndrome did not change.

Serum levels of ACTH-8AM (*p* 0.0001), cortisol-8AM (*p* < 0.0001) and ACTH-3PM (*p* 0.0383) and 24 h urinary free-cortisol excretion (*p* < 0.0001) were significantly reduced at the end of BWRP, with the exception of serum levels of cortisol-3PM, which significantly increased (*p* < 0.0001).

BWRP induced a significant hypermethylation of *clock* (*p* 0.0489) and *cry*2 (*p* 0.0102) genes, together with a significant hypomethylation of the *per2* gene (*p* 0.0307), with unchanged post-BWRP methylation status of *arntl*, *cry*1, *per1* and *per3* genes (Table 2).

### 3.3. Effects of the BWRP in Females and Males

**Figure 1:**
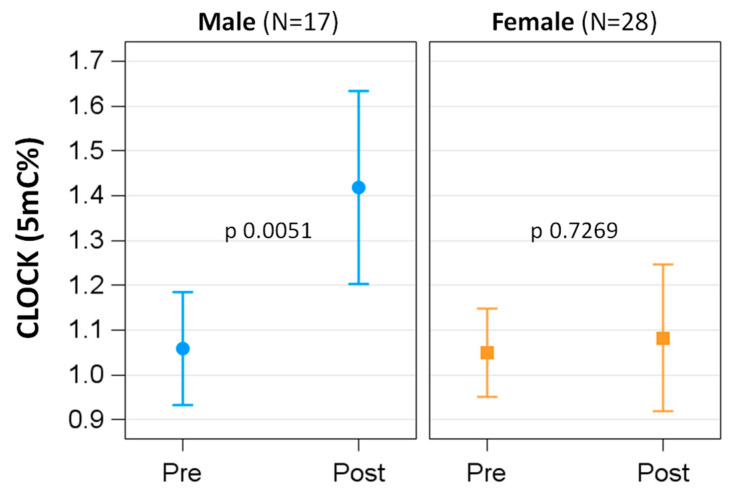
DNA methylation level of the *clock* gene in males and females: pre- vs. post-BWRP comparisons.

Gender modified the effect of BWRP on most demographic, lifestyle, biochemical and clinical parameters (Table 3). In particular, females showed a smaller change than males, except for HR, BP, glucose and CRP. When considering the methylation status of the single *clock* genes, at T0, the methylation level of *per2* was significantly higher in females than in males (F vs. M: 80.47 ± 0.63 vs. 78.31 ± 0.82, *p* 0.0445), whereas the BWRP induced a significant hypermethylation only of the *clock* gene in the male group (*p* of interaction term time × sex 0.0386; in the male group, T0 vs. T1: 1.06 ± 0.06 vs. 1.42 ± 0.11, *p* 0.0051) (Figure 1). The methylation of the other genes was not associated with different behavior between males and females.

### 3.4. Effects of the BWRP in Subjects with and without Metabolic Syndrome

**Figure 2:**
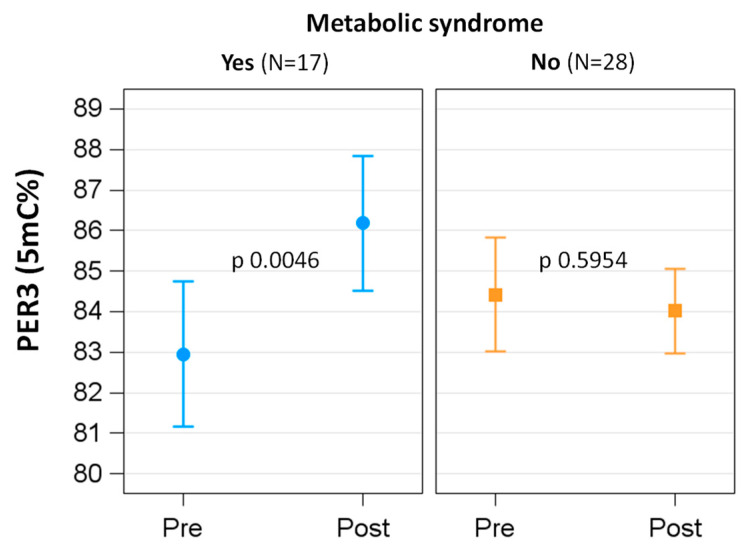
DNA methylation level of the *per3* gene in subjects with or without metabolic syndrome: pre- vs. post-BWRP comparisons.

Metabolic syndrome modified the effect of BWRP on most demographic, lifestyle, biochemical and clinical parameters (Table 4). When considering the methylation status of the single *clock* genes, whereas at T0, there were no significant differences in methylation level of any *clock* genes, the BWRP induced a significant hypermethylation only of the *per3* gene in the group with metabolic syndrome (p of interaction term time × metabolic syndrome 0.0095; in the group with metabolic syndrome, T0 vs. T1: 82.95 ± 0.89 vs. 86.19 ± 0.82, *p* 0.0046) (Figure 2).

### 3.5. Associations of Methylation Level of Clock Genes with Other Parameters

Neither the CASQ total score, MEQSA total score nor MEQSA chronotype (at T0) were associated with the methylation level of any *clock* genes. On the contrary, sleep time during a weekday (at T0) was significantly associated with the methylation levels of *arntl* (β = −0.0025, SE = 0.001 and *p* 0.0224) and *per1* (β = −0.0028, SE = 0.001 and *p* 0.0137) genes. Finally, in contrast to the CASQ alertness score, the CASQ sleepiness score (at T0) was significantly associated with the methylation level of the *cry*1 gene (β = 0.0360, SE = 0.0169 and *p* 0.0397).

Before BWRP, cortisol-3PM was significantly associated with the methylation level of the *clock* gene (β = −0.0386, SE = 0.0159 and *p* 0.0198) and *per3* (β = 0.5597, SE = 0.2303 and *p* 0.0197), whereas there was a significant association between ACTH-3PM and the methylation level of the *per1* gene (β = 0.0386, SE = 0.0141 and *p* 0.0095). Finally, ACTH-8AM and the methylation level of the *cry*1 gene were significantly associated (β = 0.0081, SE = 0.0040 and *p* 0.0489). After BWRP, there were significant associations between ACTH-8AM and the methylation level of the *per3* gene (β = 0.0464, SE = 0.0186 and *p* 0.0169) or the *clock* gene (β = 0.0054, SE = 0.0026 and *p* 0.0473).

Before BWRP, the methylation level of the *arntl* gene was significantly associated with CRP (β = -0.5526, SE = 0.2254 and *p* 0.00188), whereas that of the *clock* gene was associated with SBP (β = 0.0104, SE = 0.0030 and *p* 0.0015); that of the *cry*1 gene was associated with FM kg (β = 0.0231, SE = 0.0089 and *p* 0.0133) and REE (β = 0.0008, SE = 0.0003 and *p* 0.00135); that of the *cry*2 gene was associated with FFM kg (β = 0.0186, SE = 0.0090 and *p* 0.0433); that of the *per1* gene was associated with CRP (β = -0.4876, SE = 0.2381 and *p* 0.047); that of the *per2* gene was associated with T-C (β = -0.0440, SE = 0.0161 and *p* 0.0095) and LDL-C (β = −0.0466, SE = 0.0169 and *p* 0.0089); and that of the *per3* gene was associated with HR (β = 0.0744, SE = 0.0344 and *p* 0.0366), FFM % (β = 0.2575, SE = 0.1141 and *p* 0.0295) and FM % (β = −0.2579, SE = 0.1141 and *p* 0.0293). After BWRP, the methylation level of the *cry*2 gene was significantly associated with FM % (β = −0.0202, SE = 0.0089 and *p* 0.0290), whereas that of the *per1* gene was associated with FM kg (β = -0.0155, SE = 0.0070 and *p* 0.0330) and T-C (β = −0.0609, SE = 0.0275 and *p* 0.0327); finally, that of *per2* was associated with DBP (β = -0.1919, SE = 0.0943 and *p* 0.0485).

For further details, the reader may consult the Supplemental Material (i.e., Tables S2 and S3).

## 4. Discussion

Chronobiological misalignment has been associated with obesity and its related comorbidities, including metabolic syndrome [45]. Massive exposure to chronodisruptors (e.g., sleep deprivation, living under constant artificial light, erratic and frequently changeable eating times, nocturnal snacking, etc.) has been invoked as a reason for the widespread prevalence of pediatric obesity, particularly in Western countries [46].

In the present study, in a cohort of obese adolescents, we investigated the effects of a short-term BWRP on the DNA methylation status of a series of *clock* genes, namely *clock*, *arntl*, *cry*1-2 and *per1*-3, which, at the molecular level, regulate the circadian rhythms in SCN neurons (master *clock*) and many peripheral organs (peripheral *clocks*), such as the liver, muscles, adipose tissue and even leucocytes, the latter being the source of the extracted DNA used in our epigenetic analyses [47]. Although this experimental condition (i.e., leucocyte-based source) may actually represent a limitation of the present study, hampering the derivation of definitive conclusions, the fact that leukocytes are recognized as a “peripheral biomarker” in many fields of clinical research, including obesity [48], as well as the simplicity of blood sampling against the invasiveness of tissue biopsy (not allowed by our Ethical Committee), provides support for the present choice.

Before admission to the 3-week BWRP, obese adolescents enrolled in this study were chronobiologically characterized using validated questionnaires to quantify chronodisruption, such CASQ, MESQA and total weekly sleep score [39,40,41]. Interestingly, the methylation level of some *clock* genes, particularly *arntl*, *cry*1 and *per1*, was associated with chronodisruption, i.e. hypermethylation of these *clock* genes with decreased sleep time and increased sleepiness, indicating that our epigenetic approach was (somewhat) methodologically adequate to evaluate the relationship between weight loss and chronodisruption.

At the end of the 3-week BWRP, a hypermethylation of *clock* and *cry*2 genes occurred, as well as a hypomethylation of the *per2* gene. The epigenetic changes in the *clock* gene system were related to BWRP-induced favorable cardiometabolic outcomes, such as weight loss with WC and FM decreases, improved glucometabolic homeostasis, antidyslipidemic effects and cardiovascular benefits.

The design of our clinical study does not allow us to establish whether epigenetic changes in *clock* genes actually represent the cause or the effect of the global cardiometabolic improvement. In our opinion, the final effect of our BWRP, entailing hypocaloric diet, exercise and psychological support, appears to a sort of ”metabolic chrono-resynchronization” [49]. This is particularly evident when considering the increasing number of associations (statistically significant or close the statistical significance) of the methylation level of *clock* genes with cardiometabolic outcomes at the end of the BWRP, including FM (a surrogate of WC) (*cry*2 and *per1*), HDL-C (*cry*1), SBP (*per2*), DBP (*per2*) and TG (*per2*), which (non-surprisingly) represent the IDF criteria for metabolic syndrome [37] and (surprisingly) were not present before BWRP administration (i.e., T0).

In the present study, hypermethylation of the *per3* gene was observed at the end of the BWRP only in the group with metabolic syndrome. Among the cardiometabolic outcomes that did not change in obese adolescents without metabolic syndrome, we might invoke REE, FFM and ACTH-3PM as potential causative factors of the missing post-BWRP hypermethylation of the *per3* gene in this group. Being limited the number of the cardiometabolic outcomes that were evaluated in the present study, any attempt to explain the post-BWRP epigenetic differences among obese adolescents with or without metabolic syndrome might be too speculative. Further studies are thus needed to solve this issue, which might be of clinical interest due to the need of a “biomolecular marker” of BWRP effectiveness in metabolic syndrome, a condition that is more difficult to treat compared to essential obesity [50]. Importantly, the BWRP-induced hypermethylation of the *per3* gene might be interpreted as a negative outcome due to the post-BWRP decrease in REE and FFM in the group with metabolic syndrome, which would clinically indicate energy storage and muscle protein waste [51].

A sexual dysmorphism in body *clock* has been reported in humans, explaining, at least in part, the differing chronobiology between female and male metabolism and behavior, including the well-known eveningness preferences of men relative to women [52]. In the present study, whereas we observed hypomethylation of the *per2* gene in the male rather than female group before the BWRP, obese males showed a post-BWRP hypermethylation of the *clock* gene, an epigenetic change that did not occur in the female group.

Whereas mRNA levels of the *clock* gene were not evaluated in an in vitro study using subcutaneous and visceral adipose tissues from obese men and women, in whom a different gene expression of *per2*, *cry*1 and *bmal*1 (*arntl*) was instead found [53], in an animal model, the effects of calorie restriction on circadian rhythms in hepatic *rev-erb-α*, *ror*-γ (retinoic acid receptor-related orphan receptor-γ) and both *cry*1 and *cry*2 gene expression were demonstrated to be sex-dependent, with the exception of that of *per1*-3 genes [54]. Apart from these non-epigenetic studies, to the best of our knowledge, no study has evaluated BWRP-induced epigenetic changes in *clock* genes in obese adolescents with a sex-centered approach to date. Based on the results of the present study, we are unaware whether sex-related differences in epigenetic regulation of *clock* genes may explain the lower post-BWRP weight loss that is generally observed in obese females relative to males [55]. Because strong evidence supports the view that estrogens can modulate the expression of *clock* genes, an effect that is essential for orchestration of (even sex-related) circadian rhythms by SCN [56], a different sex-tailored BWRP might be customized, e.g., a more “chrono-resynchronizing” BWRP in females relative to males or, alternatively, a chrononutrition specific to women and men (see below).

Obesity, particularly that characterized by massive visceral adiposity, is reportedly associated with hypercortisolism, including increased 24 h urinary free cortisol excretion, cortisol secretion rate, plasma cortisol response to ACTH (or corticotropin-releasing hormone [CRH]) and salivary cortisol peak after mental stress [57,58,59,60]. Furthermore, plasma levels of cortisol-binding globulin (CBG) are lower in subjects with obesity and insulin resistance, explaining, at least in part, increased free cortisol under conditions of reduced negative feedback, such as stress [61]. Finally, adipose tissue may generate (active) cortisol from (inactive) cortisone via the 11β-hydroxysteroid dehydrogenase 1 (11β-HSD1) enzyme [62].

In the present study, the BWRP was able to tone down HPA function as documented by the decreased values of ACTH-8AM, cortisol-8AM, ACTH-3PM and 24 h urinary free-cortisol excretion at the end of our 3-week BWRP. Although conflicting results have been reported in calorically restricted obese subjects when evaluating their HPA function, a full discussion of this discrepancy is outside the scope of the present study [63].

On the contrary, this study highlights the BWRP-induced changes in the associations of some markers of HPA function with the methylation status of specific *clock* genes. For instance, pre-BWRP cortisol-3PM and ACTH-3PM were associated with the methylation status of *clock* and *per1* genes, respectively. These were associations that, interestingly, were missing at the end of the intervention, when, instead, the association of ACTH-8AM with the methylation status of *per3* was evident.

Whereas the pulsatile release of ACTH and cortisol is controlled by a negative feedback loop involving glucocorticoid receptor (GR) signaling in the hippocampus up to pituitary [64,65], circadian HPA rhythm is regulated by *clock*-gene-mediated mechanisms operating at different levels: (i) the endocrine HPA axis itself, (ii) SCN-controlled autonomic innervation and (iii) local adrenocortical circadian *clocks* [64,66].

Given that the DNA source of our epigenetic analyses was represented by peripheral leucocytes, which may not completely correspond to that from the HPA axis (e.g., pituitary or adrenals), the circadian rhythms of cortisol secretion and white blood cell count are, under physiological conditions, perfectly synchronized [67], implying common molecular mechanisms of circadian rhythmicity. Therefore, because *clock* and *per1* are the *clock* genes with a methylation level that did change at the end of the intervention (in the entire subject population, male group or metabolic syndrome) and which are implicated in associations with HPA activity, our hypothesis is that the BWRP-induced “chrono-resynchronization” involved not only metabolism in se (see above) but also the endocrine system, namely the HPA axis. Further studies are needed to further explore the relationships between *clock* genes and effects of energy restriction on HPA activity in obesity, including human and animal models [63].

The present study is subject to some limitations.

First of all, evaluation of epigenetic remodeling (i.e., levels of DNA methylation in a specific gene) does not permit a complete definition of gene/protein expression. The *clock* gene system is regulated at different levels: genetic, epigenetic, translational and post-translational [47,68]. Therefore, some BWRP-induced effects on (final) gene/protein (e.g., *clock*/CLOCK) expression might have occurred but not been detected due to our methodological approach. These considerations may also explain the difficulty encountered in interpreting our results using genetically modified animal models (e.g., *clock* mutant mice), in which obesity and dysmetabolism represent a phenotype [9].

Second, given the clinical nature of this study, the BWRP-related molecular factor(s) that could have interfered with DNA methylation machinery (e.g., DNA methyltransferases/demethylases) are unknown. Some metabolites have been demonstrated to affect DNA methylation, such as some lipids, which, in the present study, decreased after the BWRP (e.g., T-C, TG or NEFA) [22]. Metabolomics studies might be useful to answer this question and permit the selection of so-called “bioactive” nutrients to be inserted in patient diets in order to achieve nutrition-based epigenetic remodeling [69]. Alternatively, we cannot rule out that the BWRP-induced metabolic effects are mediated by PPARs and PGC 1α, which, binding (even endogenous) lipidic ligands, have been recognized as modulators of *arntl*/ARNTL and *clock*/CLOCK [4].

Third, epigenetic remodeling of *clock* genes has been associated with obesity and other metabolic disorders [30], but changes in DNA methylation of *clock* genes might be a simple epiphenomenon with no causative implications. Therefore, other (unknown or yet to be investigated) molecular mechanisms might underlie the relationships from chronobiological misalignment and obesity to BWRP-induced benefits and weight loss.

## 5. Conclusions

A short-term (3-week) BWRP administered to a chronodisrupted pediatric obese population is capable of producing beneficial cardiometabolic effects, as well as an epigenetic remodeling of specific *clock* genes, suggesting the occurrence of a post-BWRP metabolic and endocrine “chrono-resynchronization”, which might represent a “biomolecular” predictor of successful antiobesity intervention.

## Figures and Tables

**Table 1:** Demographic, lifestyle, biochemical and clinical characteristics of study participants: comparison pre vs. post BWRP (N = 45).

Characteristics	Pre	Post	*p*-Value	FDR *p*-Value
Age, years	15.8 ± 1.4		-	-
Gender				
Males	17 (37.8%)		-	-
Females	28 (62.2%)			
Smoking status				
Yes	8 (17.8%)		-	-
No	37 (82.2%)			
BMI, kg/m^2^	37.5 (35.7;39.3)	36.0 (34.2;37.8)	<0.0001	<0.0001
BMI SDS	2.94 (2.76;3.12)	2.82 (2.64;3)	<0.0001	<0.0001
WC, cm	114.8 (109.9;119.7)	110.2 (105.4;115.1)	<0.0001	<0.0001
50 Hz ohm	549.3 (529.3;569.2)	554.33 (534.4;574.3)	0.0018	0.002
FFM, kg	57.1 (53.9;60.4)	55.24 (52.04;58.45)	<0.0001	<0.0001
FFM, %	55.0 (53.0;57.1)	55.47 (53.43;57.52)	0.0008	0.0009
FM, kg	47.9 (43.4;52.4)	44.82 (40.29;49.35)	<0.0001	<0.0001
FM, %	45.0 (43.1;46.8)	43.7 (41.83;45.56)	<0.0001	<0.0001
REE, kcal	1884 (1788;1981)	1853 (1756;1950)	0.0577	0.0598
NEFA, mmol/L	0.88 (0.78;0.99)	0.7 (0.59;0.81)	<0.0001	<0.0001
Heart rate, bpm	83.3 (79.8;86.8)	77.69 (74.17;81.21)	<0.0001	<0.0001
SBP, mmHg	128.6 (126.0;131.1)	119.44 (116.88;122.01)	<0.0001	<0.0001
DBP, mmHg	79.22 (77.84;80.61)	74.44 (73.06;75.83)	<0.0001	<0.0001
MAP, mmHg	95.67 (94.05;97.28)	89.44 (87.83;91.06)	<0.0001	<0.0001
Glucose, mmol/L	4.97 (4.74;5.2)	4.63 (4.4;4.86)	<0.0001	<0.0001
Insulin, mU/L	24.32 (21.58;27.06)	17.14 (14.4;19.88)	<0.0001	<0.0001
HbA1c, mmol/L	5.41 (5.19;5.63)	5.2 (4.97;5.42)	<0.0001	<0.0001
HOMA-IR	5.39 (4.61;6.17)	3.58 (2.8;4.36)	<0.0001	<0.0001
Metabolic syndrome				
Yes	17 (37.8%)	12 (26.7%)	0.2594	0.2597
No	28 (62.2%)	33 (73.3%)		
T-C, mg/dL	157.04 (150.14;163.95)	134.18 (127.27;141.09)	<0.0001	<0.0001
HDL-C, mg/dL	43.96 (41.37;46.54)	38.36 (35.77;40.94)	<0.0001	<0.0001
LDL-C, mg/dL	100.38 (93.88;106.88)	83.73 (77.23;90.24)	<0.0001	<0.0001
TG, mg/dL	122.2 (107.32;137.08)	101.11 (86.23;115.99)	<0.0001	<0.0001
CRP, mg/dL	0.48 (0.37;0.59)	0.32 (0.21;0.43)	<0.0001	<0.0001
Cortisol-8AM, μg/L	16.06 (14.89;17.23)	14.74 (13.57;15.91)	<0.0001	<0.0001
Cortisol-3PM, μg/L	6.26 (5.41;7.1)	6.88 (6.03;7.72)	<0.0001	<0.0001
Urinary cortisol (24 h), µg	101.58 (86.78;116.37)	75.16 (60.34;89.98)	<0.0001	<0.0001
ACTH-8AM, ng/L	48.26 (41.76;54.76)	43.57 (37.07;50.07)	0.0001	0.0001
ACTH-3PM, ng/L	17.9 (16.09;19.71)	17.43 (15.62;19.24)	0.0383	0.0411
ALT, U/L	21 [13;32]		-	-
Creatinine, mg/dL	0.69 ± 0.11		-	-
CASQ score	37.7 ± 5.6		-	-
CASQ alertness score	16.4 ± 4.3		-	-
CASQ sleepiness score	21.3 ± 5.8		-	-
Weekly sleep score, min	435 ± 72		-	-
Sleep time during weekdays, min	407 ± 78		-	-
Sleep time during weekend days, min	504 ± 105			
MEQSA score	46.1 ± 8.3		-	-
Chronotype MEQSA score				
Morning (16–41)	2 (4.4%)		-	-
Intermediate (42–58)	30 (66.7%)		-	-
Evening (59–86)	13 (28.9%)		-	-

In descriptive statistics, data with normal distribution are expressed as mean  ±  standard deviation. When not normally distributed, values are expressed as median (Q1, Q3). Categorical data are reported as frequencies and percentage. We applied linear regression models for paired data to evaluate the associations between time (pre/post BWRP) and continuous variables; for categorical variables, we applied the McNemar test for paired data.

**Table 2:** Gene-specific *clock* methylation means (pre and post 3-week BWRP).

Gene methylation	TIME	Mean	(95% CI)	*p*-Value
*arntl*	pre	0.9	(0.77;1.03)	0.0592
	post	1.1	(0.96;1.23)	
*clock*	pre	1.05	(0.95;1.16)	0.0489
	post	1.21	(1.09;1.32)	
*cry1*	pre	1.49	(1.31;1.67)	0.5149
	post	1.58	(1.4;1.75)	
*cry2*	pre	1.13	(1.03;1.22)	0.0102
	post	1.31	(1.21;1.41)	
*per1*	pre	0.9	(0.76;1.05)	0.6478
	post	0.96	(0.82;1.11)	
*per2*	pre	79.83	(78.8;80.85)	0.0307
	post	79.33	(78.3;80.36)	
*per3*	pre	83.87	(82.84;84.89)	0.2493
	post	84.62	(83.58;85.67)	

We applied linear mixed regression models for paired data to evaluate associations between time (pre/post BWRP) and *clock* gene methylation. Mean and 95% CI are reported.

**Table 3:** Demographic, lifestyle, biochemical and clinical characteristics of study participants: pre- vs. post-BWRP comparison in males and females.

	Male (n = 17)				Female (n = 28)				*p*-Value of Interaction	FDR *p*-Value of Interaction
Characteristics	Pre	Post	Post-Pre	*p*-Value	Pre	Post	Post-Pre	*p*-Value		
	Mean (95% CI)	Mean (95% CI)	β ± SE		Mean (95% CI)	Mean (95% CI)	β ± SE			
BMI, kg/m^2^	38.02 (35.05;40.98)	36.33 (33.36;39.29)	−1.69 ± 0.03	<0.0001	37.17 (34.85;39.48)	35.77 (33.46;38.08)	−1.39 ± 0.03	<0.0001	<0.0001	0.0003
BMI SDS	3.1 (2.81;3.4)	2.88 (2.59;3.17)	−0.22 ± 0.01	<0.0001	2.85 (2.62;3.08)	2.78 (2.55;3.01)	−0.07 ± 0.01	<0.0001	<0.0001	0.0003
WC, cm	123.59 (116.16;131.01)	117.18 (109.75;124.6)	−6.41 ± 0.48	<0.0001	109.43 (103.64;115.21)	106.04 (100.25;111.82)	−3.39 ± 0.37	<0.0001	<0.0001	0.0003
50 Hz ohm	511.06 (481.74;540.38)	516.64 (487.3;545.98)	5.58 ± 2.52	0.0321	572.46 (549.62;595.31)	577.21 (554.37;600.06)	4.75 ± 1.9	0.0166	0.7934	0.7934
FFM, kg	66 (62.02;69.98)	64.18 (60.19;68.16)	−1.82 ± 0.21	<0.0001	51.78 (48.68;54.88)	49.82 (46.72;52.92)	−1.95 ± 0.16	<0.0001	0.6179	0.6654
FFM, %	58.78 (55.8;61.76)	59.77 (56.79;62.75)	0.99 ± 0.21	<0.0001	52.74 (50.42;55.06)	52.89 (50.56;55.21)	0.15 ± 0.16	0.3536	0.0023	0.0047
FM, kg	48.12 (40.67;55.58)	44.96 (37.5;52.42)	−3.16 ± 0.23	<0.0001	47.79 (41.98;53.6)	44.73 (38.92;50.54)	−3.06 ± 0.18	<0.0001	0.7272	0.7541
FM, %	41.21 (38.48;43.94)	40.22 (37.49;42.95)	−0.99 ± 0.24	0.0002	47.26 (45.14;49.39)	45.81 (43.69;47.94)	−1.45 ± 0.18	<0.0001	0.1355	0.1807
REE, kcal	2067 (1921;2213)	2002 (1856;2148)	−65.06 ± 25.84	0.0159	1775 (1663;1888)	1765 (1652;1877)	−10.56 ± 20.26	0.6051	0.1047	0.1494
NEFA, mmol/L	1.15 (0.97;1.32)	0.64 (0.47;0.82)	−0.5 ± 0.04	<0.0001	0.73 (0.59;0.86)	0.72 (0.59;0.86)	0.01 ± 0.03	0.9972	<0.0001	0.0003
Heart rate, bpm	76.94 (71.48;82.4)	74.12 (68.66;79.58)	−2.82 ± 0.93	0.0039	87.14 (82.89;91.4)	79.86 (75.6;84.11)	−7.29 ± 0.72	<0.0001	0.0005	0.0011
SBP, mmHg	127.06 (122.84;131.28)	120.29 (116.07;124.52)	−6.76 ± 0.84	<0.0001	129.46 (126.17;132.75)	118.93 (115.64;122.22)	−10.54 ± 0.65	<0.0001	0.0010	0.002
DBP, mmHg	79.71 (77.44;81.97)	75.29 (73.03;77.56)	−4.41 ± 0.48	<0.0001	78.93 (77.16;80.69)	73.93 (72.16;75.69)	−5 ± 0.37	<0.0001	0.3355	0.3915
MAP, mmHg	95.49 (92.84;98.14)	90.29 (87.64;92.94)	−5.2 ± 0.5	<0.0001	95.77 (93.71;97.84)	88.93 (86.86;90.99)	−6.85 ± 0.39	<0.0001	0.0121	0.0211
Glucose, mmol/L	4.94 (4.56;5.31)	4.72 (4.35;5.1)	−0.22 ± 0.07	0.0022	4.99 (4.7;5.28)	4.58 (4.29;4.87)	−0.42 ± 0.05	<0.0001	0.0209	0.0344
Insulin, mU/L	27.44 (23.01;31.86)	18.85 (14.44;23.25)	−8.59 ± 0.56	<0.0001	22.59 (19.16;26.02)	16.11 (12.68;19.54)	−6.48 ± 0.4	<0.0001	0.0038	0.0071
HbA1c, mmol/L	5.35 (4.99;5.72)	5.15 (4.78;5.51)	−0.21 ± 0.02	<0.0001	5.44 (5.16;5.73)	5.22 (4.94;5.51)	−0.22 ± 0.01	<0.0001	0.5821	0.652
HOMA-IR	5.59 (4.32;6.87)	4 (2.73;5.28)	−1.59 ± 0.18	<0.0001	5.25 (4.26;6.25)	3.32 (2.33;4.32)	−1.93 ± 0.14	<0.0001	0.1481	0.1884
Metabolic syndrome										
Yes	6 (35.3%)	4 (23.5%)	-	0.4516	11 (39.3%)	8 (28.6%)	-	0.3972	-	-
No	11 (64.7%)	13 (76.5%)			17 (60.7%)	20 (71.4%)				
T-C, mg/dL	157.12 (145.79;168.44)	128.65 (117.32;139.97)	−28.47 ± 1.16	<0.0001	157 (148.18;165.82)	137.54 (128.71;146.36)	−19.46 ± 0.91	<0.0001	<0.0001	0.0003
HDL-C, mg/dL	39.82 (36.01;43.64)	32.88 (29.07;36.7)	−6.94 ± 0.35	<0.0001	46.46 (43.49;49.44)	41.68 (38.7;44.65)	−4.79 ± 0.27	<0.0001	<0.0001	0.0003
LDL-C, mg/dL	103.88 (93.19;114.57)	82.94 (72.25;93.63)	−20.94 ± 1.14	<0.0001	98.25 (89.92;106.58)	84.21 (75.88;92.55)	−14.04 ± 0.89	<0.0001	<0.0001	0.0003
TG, mg/dL	131.18 (106.74;155.61)	101.76 (77.33;126.2)	−29.41 ± 2.07	<0.0001	116.75 (97.71;135.79)	100.71 (81.68;119.75)	−16.04 ± 1.61	<0.0001	<0.0001	0.0003
CRP, mg/dL	0.34 (0.16;0.51)	0.34 (0.17;0.52)	0.01 ± 0.02	0.7772	0.57 (0.43;0.71)	0.3 (0.17;0.44)	−0.27 ± 0.02	<0.0001	<0.0001	0.0003
Cortisol-8AM, μg/L	15.55 (13.74;17.35)	11.86 (10.06;13.67)	−3.68 ± 0.36	<0.0001	16.37 (14.96;17.77)	16.49 (15.08;17.9)	0.12 ± 0.28	0.6709	<0.0001	0.0003
Cortisol-3PM μg/L	4.66 (3.43;5.9)	5.12 (3.89;6.35)	0.46 ± 0.19	0.0174	7.23 (6.27;8.18)	7.94 (6.98;8.9)	0.71 ± 0.14	<0.0001	0.2835	0.3452
Urinary cortisol (24 h), µg	102.22 (78.21;126.23)	59.17 (35.06;83.28)	−43.05 ± 4.58	<0.0001	100.85 (82.09;119.6)	84.64 (65.88;103.4)	−16.21 ± 3.59	0.0001	<0.0001	0.0003
ACTH-8AM, ng/L	53.84 (43.28;64.41)	46.8 (36.23;57.37)	−7.04 ± 1.81	0.0003	44.87 (36.64;53.1)	41.61 (33.38;49.84)	−3.26 ± 1.41	0.0256	0.1067	0.1494
ACTH-3PM, ng/L	20.14 (17.33;22.95)	20.29 (17.48;23.1)	0.15 ± 0.36	0.6728	16.55 (14.36;18.74)	15.69 (13.5;17.88)	−0.85 ± 0.28	0.004	0.0327	0.0508

In descriptive statistics, normally distributed data are expressed as mean ± standard deviation. When not normally distributed, values are expressed as median (Q1, Q3). Categorical data are reported as frequencies and percentage. We applied linear mixed regression models for paired data to evaluate the modifier effect of sex on associations between time (pre/post BWRP) and continuous variables; for categorical variables, we applied the McNemar test for paired data.

**Table 4:** Demographic, lifestyle, biochemical and clinical characteristics of study participants: pre- vs. post-BWRP comparison in subjects with (MetS+) or without (MetS-) metabolic syndrome.

	MetS+ (n = 17)				MetS− (n = 28)				*p*-Value of Interaction	FDR *p*-Value of Interaction
Characteristics	Pre	Post	Post-Pre	*p*-Value	Pre	Post	Post-Pre	*p*-Value		
	Mean (95% CI)	Mean (95% CI)	β ± SE		Mean (95% CI)	Mean (95% CI)	β ± SE			
BMI, kg/m^2^	37.5 (35.7;39.3)	35.8 (34.0;37.7)	−1.68 ± 0.04	<0.0001	37.45 (35.65;39.26)	36.03 (34.22;37.84)	−1.43 ± 0.03	<0.0001	<0.0001	0.0002
BMI SDS	2.83 (2.64;3.02)	2.71 (2.52;2.91)	−0.12 ± 0.02	<0.0001	3.01 (2.82;3.2)	2.86 (2.67;3.04)	−0.16 ± 0.01	<0.0001	0.0565	0.0659
WC, cm	113.6 (108.5;118.6)	110.8 (105.7;115.8)	−2.79 ± 0.57	<0.0001	115.52 (110.57;120.47)	110.05 (105.12;114.99)	−5.46 ± 0.37	<0.0001	0.0004	0.0008
50 Hz ohm	555.05 (534.37;575.73)	563.46 (542.3;584.63)	8.42 ± 3.04	0.0084	545.76 (525.49;566.02)	551.11 (530.94;571.28)	5.35 ± 1.9	0.0074	0.4083	0.4234
FFM, kg	55.8 (52.53;59.07)	54.43 (51.14;57.72)	−1.37 ± 0.25	<0.0001	57.97 (54.72;61.22)	55.55 (52.3;58.8)	−2.42 ± 0.15	<0.0001	0.0010	0.0017
FFM, %	53.76 (51.72;55.8)	54.8 (52.73;56.87)	1.04 ± 0.25	0.0001	55.79 (53.78;57.8)	55.74 (53.73;57.74)	−0.05 ± 0.15	0.7349	0.0007	0.0013
FM, kg	48.74 (44.25;53.23)	45.66 (41.15;50.17)	−3.08 ± 0.28	<0.0001	47.42 (42.94;51.89)	44.51 (40.04;48.98)	−2.9 ± 0.18	<0.0001	0.6145	0.6145
FM, %	45.58 (43.7;47.46)	44.66 (42.73;46.59)	−0.92 ± 0.29	0.0029	44.61 (42.77;46.45)	43.36 (41.53;45.19)	−1.25 ± 0.18	<0.0001	0.3529	0.38
REE, kcal	1989 (1879;2100)	1875 (1755;1995)	−114.06 ± 32.39	0.0011	1827.79 (1726.85;1928.73)	1847.73 (1748.42;1947.03)	19.93 ± 19.67	0.3169	0.0013	0.0022
NEFA, mmol/L	0.86 (0.71;1)	0.77 (0.61;0.93)	−0.08 ± 0.06	0.1457	0.9 (0.78;1.02)	0.67 (0.55;0.79)	−0.23 ± 0.04	<0.0001	0.0231	0.0294
Heart rate, bpm	88.08 (84.17;91.99)	74.48 (70.3;78.66)	−13.6 ± 1.05	<0.0001	80.38 (76.74;84.02)	78.86 (75.28;82.43)	−1.53 ± 0.69	0.0323	<0.0001	0.0002
SBP, mmHg	138.02 (135.03;141.01)	128.51 (125.27;131.76)	−9.51 ± 0.92	<0.0001	122.81 (120.08;125.54)	116.15 (113.48;118.81)	−6.66 ± 0.6	<0.0001	0.0148	0.0208
DBP, mmHg	81.54 (79.93;83.15)	74.98 (73.21;76.76)	−6.56 ± 0.56	<0.0001	77.81 (76.38;79.25)	74.25 (72.85;75.64)	−3.56 ± 0.36	<0.0001	0.0001	0.0002
MAP, mmHg	100.34 (98.55;102.14)	92.8 (90.85;94.75)	−7.54 ± 0.56	<0.0001	92.83 (91.2;94.46)	88.22 (86.63;89.81)	−4.6 ± 0.37	<0.0001	0.0001	0.0002
Glucose, mmol/L	5.21 (4.95;5.48)	4.38 (4.1;4.67)	−0.83 ± 0.08	<0.0001	4.82 (4.58;5.06)	4.72 (4.49;4.96)	−0.1 ± 0.05	0.0448	<0.0001	0.0002
Insulin, mU/L	26.57 (23.69;29.46)	18.25 (15.23;21.27)	−8.32 ± 0.63	<0.0001	22.92 (20.15;25.69)	16.74 (14.01;19.47)	−6.18 ± 0.41	<0.0001	0.0084	0.0124
HbA1c, mmol/L	5.47 (5.24;5.69)	5.12 (4.89;5.35)	−0.34 ± 0.02	<0.0001	5.37 (5.15;5.6)	5.22 (4.99;5.44)	−0.16 ± 0.01	<0.0001	<0.0001	0.0002
HOMA-IR	6.24 (5.4;7.09)	3.49 (2.59;4.38)	−2.75 ± 0.21	<0.0001	4.85 (4.05;5.65)	3.61 (2.83;4.4)	−1.24 ± 0.14	<0.0001	<0.0001	0.0002
T-C, mg/dL	153.17 (145.23;161.11)	125.92 (117.73;134.11)	−27.25 ± 1.4	<0.0001	159.39 (151.7;167.09)	137.18 (129.54;144.82)	−22.21 ± 0.91	<0.0001	0.005	0.0078
HDL-C, mg/dL	42.33 (39.68;44.98)	39.65 (36.94;42.36)	−2.68 ± 0.4	<0.0001	44.94 (42.36;47.53)	37.88 (35.31;40.46)	−7.06 ± 0.26	<0.0001	<0.0001	0.0002
LDL-C, mg/dL	97.33 (89.75;104.91)	74.57 (66.75;82.39)	−22.76 ± 1.34	<0.0001	102.23 (94.88;109.57)	87.07 (79.78;94.36)	−15.16 ± 0.88	<0.0001	<0.0001	0.0002
TG, mg/dL	122.88 (106.26;139.49)	80.83 (63.87;97.79)	−42.05 ± 2.35	<0.0001	121.79 (105.5;138.07)	108.49 (92.28;124.69)	−13.3 ± 1.53	<0.0001	<0.0001	0.0002
CRP, mg/dL	0.66 (0.54;0.77)	0.4 (0.27;0.52)	−0.26 ± 0.02	<0.0001	0.38 (0.26;0.49)	0.29 (0.18;0.4)	−0.09 ± 0.02	<0.0001	<0.0001	0.0002
Cortisol-8AM, μg/L	16.22 (14.82;17.61)	15.93 (14.41;17.45)	−0.29 ± 0.45	0.5259	15.96 (14.7;17.22)	14.31 (13.08;15.54)	−1.65 ± 0.29	<0.0001	0.0173	0.0231
Cortisol-3PM, μg/L	7.25 (6.28;8.21)	7.15 (6.14;8.16)	−0.1 ± 0.22	0.65	5.66 (4.74;6.57)	6.78 (5.87;7.68)	1.12 ± 0.14	<0.0001	<0.0001	0.0002
Urinary cortisol (24 h), µg	108.94 (91.48;126.4)	92.21 (72.69;111.74)	−16.73 ± 6.15	0.0096	96.8 (80.94;112.67)	69.58 (54.09;85.07)	−27.23 ± 3.5	<0.0001	0.1549	0.1735
ACTH-8AM, ng/L	43.06 (35.31;50.8)	41.6 (33.3;49.9)	−1.46 ± 2.14	0.5005	51.42 (44.24;58.6)	44.29 (37.24;51.33)	−7.13 ± 1.4	<0.0001	0.0359	0.0437
ACTH-3PM, ng/L	16.35 (14.27;18.43)	13.52 (11.36;15.68)	−2.83 ± 0.41	<0.0001	18.85 (16.84;20.85)	18.85 (16.87;20.83)	0 ± 0.27	0.9859	<0.0001	0.0002

In descriptive statistics, normally distributed are expressed as mean ± standard deviation. When not normally distributed, values are expressed as median (Q1, Q3). Categorical data are reported as frequencies and percentages. We applied linear mixed regression models for paired data to evaluate the modifier effect of metabolic syndrome on associations between time (pre/post-BWRP) and continuous variables; for categorical variables, we applied the McNemar test for paired data. MetS+ : presence of criteria for metabolic syndrome at baseline; MetS− : lack of criteria for metabolic syndrome at baseline.

## Data Availability

The datasets used and/or analyzed in the present study are available from the corresponding author upon reasonable request.

## Supplementary Materials

The following supporting information can be downloaded at: https://www.mdpi.com/article/10.3390/ijerph192315492/s1, Table S1: Pyrosequencing assay information, Table S2: Association between chronodisruption parameters and clock gene DNA methylation, pre-BWRP; Table S3: Association between clinical and biochemical parameters and clock gene DNA methylation, pre-BWRP and post-BWRP.

## Abbreviations

11β-HSD1, 11β-hydroxysteroid dehydrogenase 1; ACTH, adrenocorticotropic hormone; ALT, alanine transaminase; ARNTL, aryl hydrocarbon receptor nuclear translocator like; ASP, aspartate transaminase; BMI, body mass index; CI, confidence interval; BMAL1, brain- and muscle-ANRT-like protein; bpm, beats per minute; CLOCK, circadian locomotor output cycles kaput; CRH, corticotropin-releasing hormone; CRP, C-reactive protein; CRY1-2, cryptochrome 1-2; DBP, diastolic blood pressure; DNAm, DNA methylation; ERB-α, reverse erythroblastosis virus-α; F, female; HDL-C, high-density lipoprotein cholesterol; gamma-GT, gamma-glutamyl transferase; HC, hip circumference; HOMA-IR, homeostatic model assessment for insulin resistance; HR, heart rate; IQR, interquartile range; LDL-C, low-density lipoprotein cholesterol; M, male; MAP, mean arterial pressure; MetS, metabolic syndrome; NPAS2, name derived from PERARNT-SIM protein-2; OR, odds ratio; PER1-3, period 1-3; PGC 1α, PPAR-γ coactivator 1α; PPAR, peroxisome proliferator-activated receptor; ROR-α, retinoic acid receptor-related orphan receptor-α; SBP, systolic blood pressure; SD, standard deviation; SE, standard error; T-C, total cholesterol; TG, triglycerides; WC, waist circumference; yr, year.
