# Supplementary material for: Changes in DNA Methylation of Clock Genes in Obese Adolescents after a Short-Term Body Weight Reduction Program: A Possible Metabolic and Endocrine Chrono-Resynchronization
Source: Int J Environ Res Public Health. 2022 Nov 22;19(23):15492. doi: 10.3390/ijerph192315492 (PMC9738941; doi:10.3390/ijerph192315492)
Supplement: Supplementary file 1 [file ijerph-19-15492-s001.zip › Table S1.pdf]

Table S1. Pyrosequencing assay information.

Gene	Chromosome Position [1]	CpG Sites		Primers: Forward (F) Reverse (R) Sequencing (S)	Sequencing Length	T° Annealing
ARNTL	chr11:13275818 -13278227	2	F	GGGGTTAGTTTGGGTAATAGAATTAG	38 bp	54 °C
			R	Bio-TAAACTCCCTAAATAAAAAAACAAC		
			S	TTATTTTATTTTATTTTAGT		
CLOCK	chr4:55547142 -55547530	2	F	TTTTTAGGAGATGGGAGAAGATGT	28 bp	51.5 °C
			R	Bio-TAAAAAATCCAAAAACCAAAAAAAA		
			S	TTTTTTGTTAATATT		
CRY1	chr12:105617622 -105618592	3	F	TTTGTGAGGGAAGGTTTAGTTT	27 bp	56 °C
			R	Bio-AACAATTTCCAAACCCTCC		
			S	TTTTTAAGGGTTATGAG		
CRY2	chr11:45846906 -45847578	4	F	TGTTTTTTGAGATTTGGTTTATTTT	33 bp	54 °C
			R	Bio- CCAAAACCCCTCTACCATTAACTA		
			S	TGTTTTTTGAGATTTGGTTTATTTT		
PER1	chr17:8151724 -8152661	3	F	TAGGGTTAGGGATTGGAGAATAGA	27 bp	52 °C
			R	Bio-ACCCAAACAAAAAACACACTATC		
			S	GGGTTAGGAGTGTAGATTTT		
PER2	chr2:238288036 -238291073	3	F	TGAGAAAGGTAGTATTTTTAAGG	34 bp	52 °C
			R	Bio-AAAACTCCACATACCCCACAC		
			S	AGGAGGTTGTTTTGGGAGAT		
PER3	chr1:7784068 -7785195	3	F	TGTTTGTTATTGATTGTAAAGTGAG	25 bp	52 °C
			R	Bio-AATTTAAATCCCCCTTTCCCTAC		
			S	TGTTTGTTATTGATTGTAAAGTGAG		
